# Exploring Priority Intrapartum Services Associated with Childbirth Experience During Vaginal Birth: A Multicenter Cross-Sectional Study

**DOI:** 10.3390/healthcare14183115

**Published:** 2026-09-21

**Authors:** Jiasi Yao, Jinbing An, Xian Zhang, Jiahe Li, Hongxiao He, Junying Li, Hong Lu, Xiaona Huang, Xiaobo Tian, Junxiao Liang, Qiong Luo, Xiu Zhu

**Affiliations:** 1 School of Nursing, Hebei Medical University, Shijiazhuang 050017, China; 18901194@hebmu.edu.cn; 2 Department of Health Informatics and Management, School of Health Humanities, Peking University, Beijing 100191, China; termison@bjmu.edu.cn (J.A.); luoqiong21@sina.com (Q.L.); 3 School of Nursing and Health, Zhengzhou University, Zhengzhou 450001, China; zhangxian@zzu.edu.cn; 4 School of Nursing, Peking University, Beijing 100191, China; jiahe@pku.org.cn (J.L.); hongxiao1128@163.com (H.H.); 18003300887@163.com (J.L.); luhong@bjmu.edu.cn (H.L.); 5 Department of Medicine and Health, Handan Vocational College of Science and Technology, Handan 056046, China; 6 Child Health and Development Section, United Nations Children’s Fund China Office, Beijing 100600, China; xhuang@unicef.org (X.H.); xtian@unicef.org (X.T.); juliang@unicef.org (J.L.)

**Keywords:** childbirth experience, random forest, Bayesian network, intrapartum service, health promotion

## Abstract

***Background*:** A woman’s childbirth experience is important to her health and family well-being. Evidence-based intrapartum care may support a positive experience. When resources are constrained, identifying services that are most strongly associated with childbirth experience may help inform quality-improvement priorities. This study aimed to explore priority intrapartum services associated with women’s experience following vaginal birth. ***Methods*:** A multicenter cross-sectional study was conducted in mainland China. A total of 498 postpartum women from ten tertiary facilities were involved. Data were collected through retrospective review of medical records and self-administered questionnaires completed by postpartum women. A self-designed questionnaire was used to collect general information and information on intrapartum services, and the Childbirth Experience Questionnaire was used to assess childbirth experiences. Random forest and Bayesian network analysis were used to examine associations and identify potentially high-priority services. ***Results*:** Childbirth experience scores ranged from 27 to 76, with a mean of 61.29. In bootstrap analyses of the random forest model, coefficient of variation for variable-importance estimates ranged from 5.1% to 32.3%. The Bayesian network contained seven structurally compact communities, five of which were closely associated with childbirth-experience outcomes; hospital type was an important connecting node between intrapartum services and childbirth experience. Among the top ten factors associated with the overall childbirth experience, several intrapartum services stood out. These included the type, initiation time, and duration of skin-to-skin contact; the number of evidence-based services received during the first and second stages of labour; and the use of pain-relief methods. ***Conclusions*:** Receiving a broader range of evidence-based intrapartum services, longer uninterrupted skin-to-skin contact and access to pain-relief options were associated with more positive childbirth experience scores.

## 1. Introduction

With the improvement in healthcare, the quality of intrapartum services is evaluated by not only the mother and newborn’s morbidity and mortality, but also a woman’s experience during childbirth [1]. Global agendas are expanding the focus of intrapartum services to ensure that women and their babies not only ‘survive’ but also ‘thrive’ from their labour and birth [2]. World Health Organization (WHO) [2] emphasized the concept of ‘positive childbirth experience’ as a critical aspect and recognized it as a significant endpoint for all women undergoing childbirth. Childbirth experience is multi-dimensional [3], comprehensive and special to women [4]. Negative childbirth experience affects women’s breastfeeding behaviours [5] and increases women’s risk of postpartum mental disorders [6].

A woman’s experience and satisfaction during labour could be improved by evidence-based intrapartum services, such as allowing a childbirth companion [7], providing pharmacological and non-pharmacological pain-relief options [8], and encouraging early skin-to-skin contact (SSC) [9]. The WHO has issued standardized service packages, including uninterrupted SSC, multi-mode pain-relief and continuous labour companionship [2]. However, in some areas in China and other similar developing countries, these services are prohibited due to lack of medical staff or equipment. Hence, it is crucial to balance the clinical needs of clients and the restricted medical resources. In-depth analyses are still required to gain deep insights into how multiple intrapartum services interact with each other in order to determine the prioritized intrapartum services critical for achieving the most benefits for women’s childbirth experience under limited medical resources.

Standard regression models may be effective in independently verifying the correlation between individual services and childbirth experience [10,11]. In contrast, some non-parametric statistical methods, such as random forest [12,13], Bayesian network [11,14,15] and community detection [16,17], may be applicable for the analysis of the influential factors and their complex relationship (linear or nonlinear). In our analysis, in combination with conventional regression approaches, we integrated random forest methods and Bayesian network methods for the following benefits. Firstly, our project involved a large number of categorical variables and had to deal with complex category combinations, and the random forest method can automatically detect effective interactions through recursive partitioning. Secondly, random forest and Bayesian network can explore the nonlinear dose–response relationship of different variables without the limitation of a specific functional form among variables. Thirdly, the Bayesian network method can automatically determine the direction of conditional dependence among variables through structure learning, which is beyond the traditional regression coefficient table. Furthermore, based on the conditional probability distribution (CPD), we can draw a candidate path map of interventions. Specifically, our network analysis comprised performing a univariate statistical analysis, evaluating and ranking factors related to childbirth experience, and constructing a Bayesian network.

Hence, this study aimed to identify the priority intrapartum services and examine their associations with women’s childbirth experience following vaginal birth using random forest and Bayesian network analyses. For this study, priority services were defined as those with the strongest statistical relationships with childbirth experience scores. The findings were intended to inform, rather than determine, clinical quality-improvement priorities.

## 2. Materials and Methods

### 2.1. Setting and Sample

We conducted a multicenter, cross-sectional study in mainland China. A purposive sampling strategy was used to select hospitals, aiming to achieve diversity across geography, hospital type and economic context. The ten tertiary hospitals comprised four general hospitals and six specialist maternity hospitals located in nine provincial-level areas. They represented eastern China (Beijing, Tianjin, Hangzhou, Shanghai, Suzhou, Guangzhou and Foshan), central China (Wuhan) and western China (Chengdu).

A time-stratified quota sampling method was used to recruit participants (see Supplementary Material S1). According to the big-sample principle, the independent sample was set at no less than 30 in every single facility [18], with the sample size determined to be at least 300 cases. Based on the principle in machine learning methods, the number of predictors in random forest and nodes in Bayesian network analysis was used to reach the sample-to-predictor/node ratio of 5:1–10:1 [11]. A total sample size of 300–500 was estimated.

Data were collected from September to October 2022. Eligible women were approached and asked about their willingness to participate in the study. Women who had undergone vaginal birth, were above the age of 18, had a single full-term living fetus, and were classified as grade 2 or below in a pregnancy risk assessment were eligible to participate in the study. Women who were unable to read or communicate, or who had serious physical or mental complication during pregnancy and childbirth, were excluded from the study. Questionnaires were distributed at 24 to 48 h postpartum to reduce long-term recall bias.

### 2.2. Data Collection

The independent variables comprised two domains: women’s basic information and the intrapartum services they received. The dependent variable was the childbirth experience score (response variable Y).

#### 2.2.1. Women’s Basic Information

This was extracted from medical notes by researchers using a case report form, including demographic variables and obstetric outcomes. Demographic information included age, educational level, household register types for the couple, occupational types, family annual incomes, parity, pregnancy complications and risk level, maternity leave and prenatal education. Obstetric outcomes comprised admission into hospital, birth mode, perineal conditions, blood loss (total), gender of newborn, and time to initiate the first instance of breastfeeding.

#### 2.2.2. Intrapartum Services

A questionnaire was developed to collect information on the intrapartum services women received. Based on a literature review of the WHO recommended intrapartum services list, which was widely adopted in the Chinese context [19], the draft of the questionnaire was developed and reviewed by experts in this field to ensure its relevance. Prior to formal data collection, a pilot test was conducted with a small sample to verify the feasibility and clarity of the questionnaire. Minor revisions were made to the wording based on feedback from participants.

In addition to this, we calculated and recorded the numbers of services each woman received during the first and second stages of labour. These summary variables were termed ‘services during the first stage of labour’ and ‘services during the second stage of labour’, respectively.

Services in the First Stage of Labour: Companion in the first stage of labour (yes, no), free mobility in the first stage of labour (yes, no), permission to eat and drink in the first stage of labour (yes, no), reminder to urinate in the first stage of labour (yes, no), and the same midwife before and during labour (yes, no, unclear).

Services in the Second Stage of Labour: Position during labour (supine, semi-recumbent, lateral position, use of birthstools and others), companion during the second stage of labour (yes, no), spontaneous pushing during the second stage of labour (yes, no), and fundal compression during the second stage of labour (yes, no).

Pain-Relief Methods: Pharmacological methods (epidural, others) and non-pharmacological methods (breathing techniques, birth ball, continuous support, water immersion, massage, aromatherapy, yoga, acupuncture, hypnosis, transcutaneous electrical nerve stimulation, others).

SSC-Related Services: Type of SSC (no, yes and naked, yes with clothes), time to initiate SSC (immediately after childbirth within one minute, within five minutes after childbirth, after five minutes after childbirth), and duration of SSC (less than 30 min, 30–59 min, 60–89 min, 90 min and above).

#### 2.2.3. Women’s Childbirth Experience

We used the Childbirth Experience Questionnaire (Chinese version) (CEQ-C) to measure women’s childbirth experience. This questionnaire was translated into Chinese and adapted to the Chinese context [20]. It comprised 19 items in four dimensions: Dimension 1, *Professional Support* (6 items); Dimension 2, *Own Capacity* (6 items); Dimension 3, *Self-Perception* (4 items); and Dimension 4, *Participation* (3 items). Each item was scored using a 4-point Likert scale ranging from 1 (totally agree) to 4 (totally disagree). This questionnaire was shown to be robust in the Chinese context with a Cronbach’s α coefficient of 0.88 [20].

### 2.3. Statistical Methods

#### 2.3.1. Basic Univariate Statistical Method

In this section, we used some descriptive statistical methods in SPSS software (version 26.0) to extract the basic information of variables. We also used Spearman coefficients to explore the correlation between some pairs of intrapartum services, which can be used to judge their positive or negative effect and help to search for better interventions. Furthermore, we used a cumulative probability curve to compare the effects of different choices on Response Y. For example, for duration of SSC (X), such as a time of less than 30 min ($x_{1}$), 30–59 min ($x_{2}$) and so on, we could obtain the cumulative probability of Score Y on $x_{i}$, as defined by $y_{t}^{i}=P(Y>t|X=x_{i})$. Theoretically, a larger probability value means the corresponding factor will tend to have larger observations, and such information may help in developing the best recommendations to improve the childbirth experience.

#### 2.3.2. Subgroup Analysis

Considering the possible effects of clustering within hospitals, we calculated Intraclass Correlation Coefficient (ICC) for the childbirth experience score using a null model with hospital ID as a random intercept. The ICC was 0.315 [τ^2^/(τ^2^ + σ^2^) = 27.61/(27.61 + 59.98) = 0.315], indicating that approximately 31.5% of the total variance in childbirth experience scores is attributable to differences between hospitals, such as facilities and the quality of services.

To formally validate the core pathways identified by the Bayesian network while accounting for hospital-level clustering (ICC = 0.315), we fitted a linear mixed-effects model with hospital-specific random intercepts, and then performed Type II Wald chi-square tests, which can assess the overall significance of predictors, using the Anova function from the “car” package.

Given that the majority of the variables were categorical, we performed a permutational multivariate analysis of variance (PERMANOVA) to characterize the heterogeneity between two types of hospitals in our analysis and to identify key variables that may explain differences in services. To account for multiple testing and control the false discovery rate (FDR), we applied the Benjamini–Hochberg procedure to all univariate *p*-values. After FDR correction, variables with FDR-adjusted *p* < 0.05 were considered significantly different between the hospital types. Besides statistical significance, we quantified the magnitude of between-group differences using effect sizes to evaluate intervention priorities. For categorical variables, we reported Cramér’s V, which ranged from 0 to 1, with larger values indicating stronger associations between the variable and hospital type.

All analyses were performed using R (version 3.4.3) with the “vegan”, “cluster”, “car” and “tidyverse” packages. Differences in service factors and outcomes between hospital types are presented descriptively. Their rankings were used to inform subsequent exploratory analyses rather than to establish intervention priorities.

#### 2.3.3. Network Analysis Part 1: Random Forest Method

The total CEQ-C score was used as the outcome (Y), and the importance of each variable was estimated and ranked using a random forest model in R [12,13]. Candidate variables entering the model were pre-specified before analysis based on WHO intrapartum guidelines, the literature and expert consultation; random forest was used to rank variable importance rather than to perform a post hoc variable screening. We evaluated the function of the selected variable by the DROP-OUT method for variable ranking in this study. Specifically, we calculated the accuracy of predicting indicator Y with or without variable X, and then evaluated the importance of X to Y by comparing the prediction results. We performed a transformation of Min-Max on all the values to make them belong to (0, 1). Transformed random forest value was calculated for each variable, with ‘0’ indicating the least effect and ‘1’ the strongest effect.

#### 2.3.4. Network Analysis Part 2: Bayesian Network

Bayesian network was constructed by use of R package ‘bnlearn’ and hill-climbing method [14]. To improve the reasonability, we added a blacklist to prohibit other nodes from pointing to demographic variables and prohibit outcome variables from pointing to other nodes. In addition, we kept the edges which had strength and direction values of at least 0.5.

Initially, based on the Bayesian Information Criterion (BIC), we discretized variables into categories in order to control the variability or bias of observations. In detail, for several candidate group numbers of K, we estimated Bayesian networks and corresponding BIC. Usually, the larger the K, the smaller the BIC. In addition, with unit variation in K, a larger change in BIC is preferable. For example, for K = 2, 3, 4, 5, we obtained four BIC values (−8477.427, −9542.371, −10,108.098, −10,826.028), and we selected K = 3, which involved a smaller K value and a larger change in BIC. As a sensitivity analysis, we further evaluated the consistency of this selection using Akaike Information Criterion (AIC) and Bayesian Dirichlet Equivalent (BDE) scores. All three criteria consistently supported the chosen discretization schemes.

Subsequently, based on survey data, the conditional probability $P\left(X_{j}|X_{i}\right)=p_{ij}$ between any two variables ($X_{i}$ and $X_{j}$) was estimated, and a conditional independence test was completed in a similar way to that of a general hypothesis test. In this way, $p_{ij}$ can be transformed into $E_{ij}=1$ or $E_{ij}=0$, which means the presence or absence of the edge between $X_{i}$ and $X_{j}$, respectively. In the end, all the $E_{ij}$ can be deemed an adjacency matrix. Furthermore, we could also obtain some long paths from $X_{i}$ and $X_{j}$, which generally represent an indirect correlation. In general, the shorter the distance between any pair of $X_{i}$ and $X_{j}$ is, the stronger the influence of $X_{i}$ and $X_{j}$. This may serve as the foundation to propose intervention strategies. Furthermore, we noted that discretization process risks information loss, and sparse data across multiple categories may produce unstable conditional probabilities and redundant network edges. To obtain a stable and reliable Bayesian network, a bootstrap method was employed with 1000 iterations, and the edges appeared in at least 50% of the bootstrap iterations that were included in the final averaged Bayesian network [21,22].

Overall, we combined the above statistical methods to provide a comprehensive factor evaluation and visual display of factor interaction. Apart from an initial ranking of the variables in random forest, the final detection of prioritized variables was decided based on the important communities and edges in the Bayesian networks, thereby gathering evidence for clinical recommendations. Note that all statistical analyses in this cross-sectional study only identified pairwise and network-level associations between variables, and no causal inference can be drawn from the present dataset.

### 2.4. Ethical Considerations

Electronic informed consent was obtained from women prior to survey commencement. Explicit information about the study aims, voluntary participation, confidentiality, and withdrawal rights was provided.

This study is reported following the STROBE guidelines [23]. The CEQ-C scale used in this study was independently translated, culturally adapted, validated by our research team and published [20]. As the developers of the Chinese adaptation, we hold full rights to use this Chinese version for academic research and publication. No further permission is required for its use in the present study.

## 3. Results

### 3.1. Basic Information of Study Sample

We invited and screened 654 women. Among them, 154 women were excluded because of high-risk pregnancy, preterm delivery, etc. (Supplementary Figure S1). A total of 500 women took the survey, and 498 women completed the questionnaire, achieving a response rate of 76.1%. In total, 302 women (60.6%) were from tertiary maternal hospitals and 196 of them (39.4%) from tertiary general hospitals. The sample had a mean age of 30.47 years (SD = 3.009). Nearly half of them were primiparous and half were multiparous women. There were no missing data in this study. Details of women’s demographic and obstetric outcomes are shown in Table 1.

### 3.2. Intrapartum Services Women Received During Childbirth

The intrapartum services women received during childbirth are illustrated in Supplementary Table S1. Women reported receiving a range of evidence-based services, including continuous support during labour, pain-relief and SSC.

Most women (N = 420, 84.3%) had a companion during the first stage of labour, whereas 259 (52.0%) had a companion during the second stage. Both pharmacological and non-pharmacological pain-relief methods were available, and some women received both. Two thirds of the women (N = 328, 65.9%) reported receiving non-pharmacological pain-relief; the remained did not receive it or were unsure. Two thirds of the women (N = 338, 67.9%) received pharmacological pain-relief services, mostly epidural analgesia (N = 322, 95.3%). Over three quarters (83.5%) of the women reported the experience of SSC service, and the duration of SSC ranged from 1 to 120 min with a mean duration of 36.52 min, with half of the women having an SSC duration of less than 30 min. The majority of the women were allowed to adopt any position they preferred, and the most adopted position was the supine position (57.0%). One third of the women were encouraged to push spontaneously during the second stage of labour.

### 3.3. Scores of Women’s Childbirth Experience

The results of the CEQ-C showed that the average score of women’s childbirth experience was 61.29, ranging from 27.00 to 76.00 (out of total score of 76 for 19 items). The quartiles were 54.00 (25.0%), 62.00 (50.0%), and 69.00 (75.0%), which meant the childbirth experience still has much room for improvement. Among the four dimensions, Dimension 1, ‘*Professional Support*’, ranked the highest, and Dimension 3 ‘*Self-Perception*’, ranked the lowest. For each item, 16 of the total 19 items scored 3 and above, with the item ‘I felt secure’ ranking the highest. Three items got scores of less than 3, which belonged to Dimension 3, ‘*Self-Perception*’, and Dimension 4, ‘*Participation*’ (Table 2).

### 3.4. Subgroup Analysis of Hospitals

As the ICC was 0.315, indicating that approximately 31.5% of the total variance in the outcome was attributable to hospital differences, we attempted to divide hospitals into two categories to perform a subgroup analysis, namely tertiary maternal hospitals and tertiary general hospitals. Under this strategy, we first compared the distributions of all key characteristics between the two hospital types using permutation-based multivariate analysis of variance (PERMANOVA) with Gower distance matrices followed by univariate comparisons with FDR correction. In this process, our method automatically selected either the chi-square test or Fisher’s exact test based on the characteristics of the observed data. Finally, we provided adjusted *p*-value, Cramer’s V values as effect sizes and ranked them based on magnitude (Table 3). This stratified descriptive approach allowed us to profile the two hospital types and identify potential targets for quality-improving interventions, which would be further explored in the Bayesian network analysis.

In the multilevel analysis, Type II Wald chi-square tests confirmed that four core service variables were significantly associated with childbirth experience after adjusting for covariates and hospital-level random effects: S2.Serve (χ^2^= 15.37, df = 1, *p* < 0.001), SSC.Duration (χ^2^= 14.87, df = 3, *p* = 0.002), SSC.type (χ^2^= 7.30, df = 2, *p* = 0.026), and Nonphamacological (χ^2^= 6.57, df = 2, *p* = 0.037). Among the covariates, Risk.level (χ^2^= 9.13, df = 2, *p* = 0.010) and Multipara (χ^2^= 6.32, df = 1, *p* = 0.012) were also significant. These findings aligned closely with the core pathways identified in the Bayesian network, cross-validating the robustness of the observed associations.

### 3.5. Prioritized Services Explored by Network Analysis

#### 3.5.1. Important Services Ranked by Random Forest Method

We calculated the transformed random forest values of influential factors of childbirth experience via the random forest method. This process was completed by the R package of “random forest”, and the hyperparameters were: ntree = 1000, mtry = 3. The top ten influential factors are shown in Table 4. The results indicated that seven out of the top ten factors related to the intrapartum services. SSC-related services, such as duration of SSC, type of SSC, and initiation time of SSC, were important factors associated with the overall childbirth experience. Moreover, the services a woman received during the two stages of labour ranked high on the list. Particularly, company during the first and second stages of labour and the non-pharmacological pain-relief methods among the intrapartum services were important factors related to childbirth experience.

To assess the reliability of the ranking results, using the out-of-bag method, we employed 100 bootstrap replicates of the random forest model and calculated the coefficient of variation (CV) for each variable’s MeanDecreaseGini. All the CV values ranged from 5.1% to 32.3%, well below the conventional threshold of 50%, indicating relatively limited variability across the bootstrap samples, although this does not establish external validity.

#### 3.5.2. Main Communities Explored by Bayesian Network

**Figure 1:**
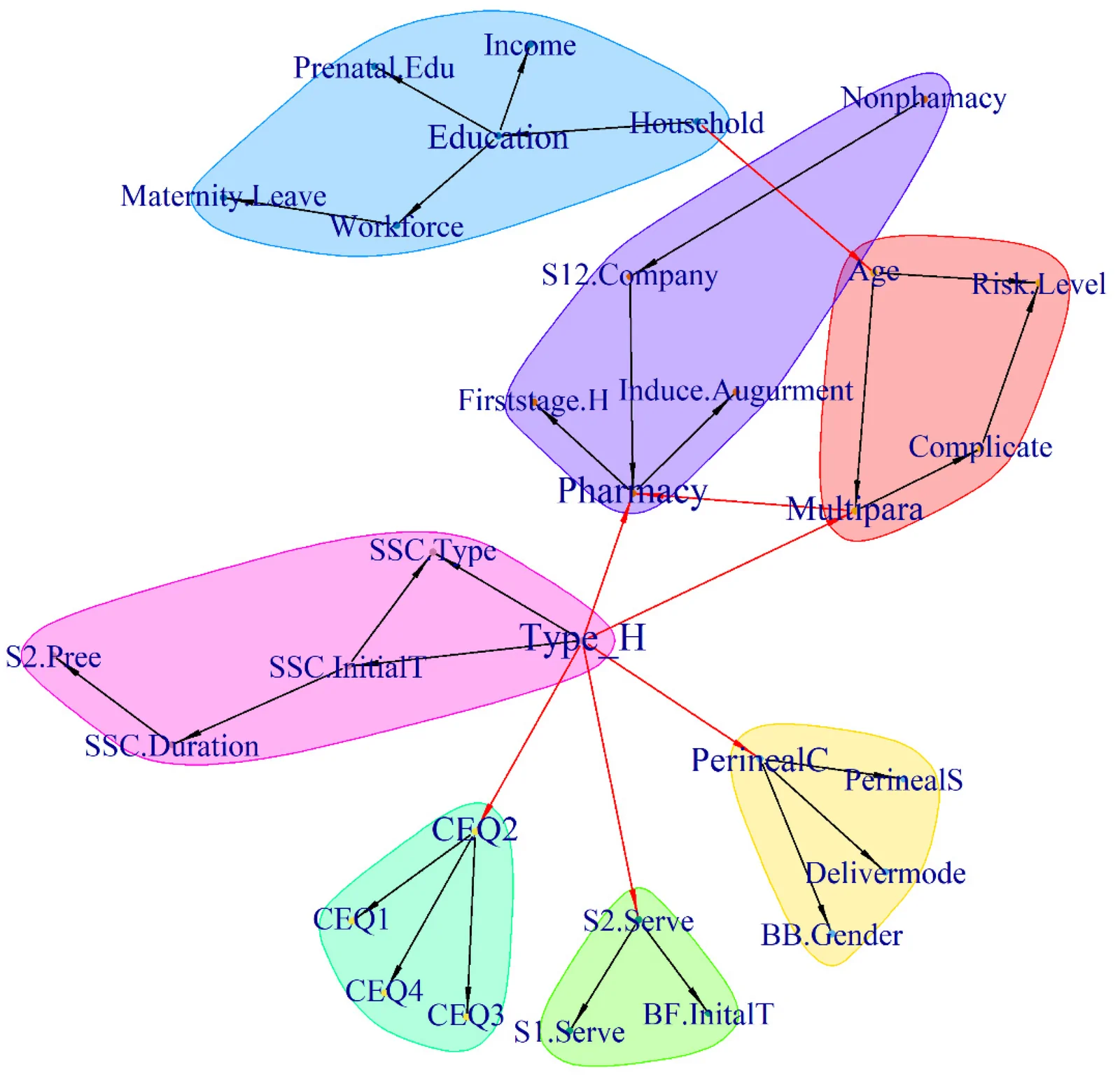
Bayesian network of factors associated with childbirth experience. CEQ1: Dimension 1, *Professional Support* of CEQ-C; CEQ2: Dimension 2, *Own Capacity* of CEQ-C; CEQ3: Dimension 3, *Self-Perception* of CEQ-C; CEQ4: Dimension 4, *Participation* of CEQ-C; S1.Serve: services during the first stage of labour; S2.Serve: services during the second stage of labour; BF.InitialT: time to initiate breastfeeding; SSC.Duration: the duration of SSC; SSC.InitialT: the time to initiate SSC; SSC.Type: SSC, whether provided while naked or not; S2.Pree: fundal compression during the second stage of labour; Type_H: type of hospital; PerinealC: perineal conditions; PerinealS: perineal suture; Delivermode: birth mode; BB.Gender: gender of newborn; Pharmacy: pharmacological pain-relief methods; Firststage.H: blood loss in the first stage of labour; Induce.Augument: induction or augmentation of labour; S12.Company: company during the first and second stage of labour; Nonpharmacy: non-pharmacological pain-relief methods; Age: women’s age, divided into three groups; Multipara: multiparous women; Complicate: complications during pregnancy and childbirth; Risk.Level: pregnancy risk level; Household: household register for the couple; Education: women’s educational level; Workforce: occupational type; Maternity.Leave: the duration of women’s maternity leave; Prenatal.Edu: prenatal education; Income: family annual income divided into groups.

The Bayesian network model was used to explore direct and indirect interactive relationships between women’s childbirth experience and the included variables. Based on the above subgroup analysis results, the type of hospital defined as Type_H was incorporated into the network, which can explore the associations of hospitals with other factors. Multiple interpretable and structurally stable community clusters were identified within the network based on the correlation and connection strength among all included variables (Figure 1). Each extracted community corresponds to a specific thematic dimension or homogeneous attribute category of childbirth-related indicators, covering core domains including SSC characteristics, maternal socio-demographic characteristics, and the four dimensions of the CEQ-C, as well as intrapartum service and pain management indicators.

Several structurally compact and functionally distinct local communities were further extracted from the overall network. Firstly, the four validated dimensions of CEQ-C formed a highly cohesive independent community with strong internal connections. These four dimensions comprehensively evaluate women’s childbirth experience from the perspective of four dimensions. The clustering of the four CEQ-C dimensions within one Bayesian network community aligns with the pre-defined four-factor structure of the scale, which provides auxiliary evidence of stable structural consistency. Secondly, SSC-related factors were found to show close connections with each other in the SSC cluster, including SSC duration, SSC implementation type, and the initial time of SSC. In this cluster, the type of hospital presented extensive associations with SSC-related indicators, which may reflect various levels of implementation of SSC services. Thirdly, a pain-relief cluster around the pain-relief options, as well as company during labour, was observed. These variables are closely related with each other regarding women’s feelings of security and the support they received during labour. Fourthly, a typical socio-demographic cluster centred on women’s educational level was observed in the network. This core variable exhibited close internal correlations with multiple socio-economic and demographic indicators. These interrelated variables jointly constitute a complete socio-demographic characteristic system for participants.

#### 3.5.3. Important Pathway Explored by Bayesian Network

The network visualization also intuitively demonstrated the complex interaction pathways between core clinical service factors and maternal childbirth experience. Note that the network edges and arrows only represent statistical correlations and could not be interpreted as causal pathways. Notably, hospital type emerged as a critical institutional factor closely intertwined with intrapartum care services and subsequent maternal childbirth experience. Variations in service implementation intensity, standardization of delivery care protocols, and clinical management norms across different types of hospitals lead to distinguishable execution levels of intrapartum services, SSC arrangements, and pain management interventions, which further contribute to individual differences in women’s childbirth experience. Specifically, intrapartum services during the second stage of labour and the initial time of SSC were identified as key bridging variables closely linked to the CEQ-C community, serving as critical influencing factors of women’s overall childbirth experience. A strong internal correlation was detected between first-stage labour services and second-stage labour services, indicating consistent service quality across different delivery stages.

Collectively, for the Bayesian network, we performed bootstrap resampling (n = 1000) to assess the stability of the network structure. For each bootstrap sample, we learned the network structure and calculated the edges’ strength and direction, and kept edges with values larger than 0.5. The Bayesian network analysis results revealed seven structurally compact local communities within the full network. Among them, five core communities were predominantly associated with women’s childbirth experience, including: (1) SSC services (initiation time, duration, and type of SSC), (2) services during the second stage of labour, (3) services during the first stage of labour, (4) pain-relief methods, and (5) as a fundamental institutional variable, hospital type, which regulates the quality of execution of various delivery care services, having a foundational impact on maternal childbirth experience.

To sum up, based on the results in Bayesian networks and random forest, seven intrapartum services were found to be the most rewarding and closely related to childbirth experience, and therefore should be considered a priority (Table 5).

### 3.6. Relationship with Women’s Childbirth Experience

To determine possible implications for clinical practice, we examined the associations between the seven selected intrapartum services and childbirth experience (Table 5). A greater number of services being received during either the first or second stage of labour was associated with higher childbirth-experience scores (*p* < 0.05). Use of non-pharmacological pain-relief was associated with higher overall CEQ-C scores and higher scores in all four dimensions (*p* < 0.05). Pharmacological pain-relief was associated with the Professional Support and Self-Perception dimensions and with the overall CEQ-C score (*p* < 0.05), but not with the other dimensions.

SSC services were shown to be closely connected with each other, and this community was directly associated with women’s childbirth experience. Direct SSC and initiation immediately after birth were each associated with higher childbirth-experience scores (*p* < 0.05) (Supplementary Figure S2). Longer SSC duration also showed a positive association with childbirth-experience scores.

We also explored the possible demographic and obstetric confounders or effect modifiers in the associations between key service variables and childbirth experience (Supplementary Tables S2–S8). Take prenatal education, for example: we first compared the utilization of key service variables between women with and without prenatal education using chi-square tests. For a confounding assessment, we compared the regression coefficients of each service variable between crude models (without prenatal education) and adjusted models (including prenatal education). A change in the coefficient exceeding 10% was considered evidence of confounding. For effect modification assessment, we tested the interaction terms Service_Variable × Prenatal.edu in separate logistic regression models.

As shown in Supplementary Table S8, adjustment for prenatal education changed the coefficients for SSC-related services and pharmacological pain-relief by less than 10%, providing little indication of confounding by prenatal education. In contrast, the coefficient for non-pharmacological pain-relief changed by 18.01%, suggesting potential confounding.

Interaction tests further confirmed that prenatal education did not significantly modify the relationship of any service variable with childbirth experience, as all interaction terms were non-significant (*p* values ranged from 0.198 to 0.810). These findings collectively indicated that the positive associations between SSC-related services and pharmacological pain-relief with childbirth experience were consistent for women with and without prenatal education, while the association between non-pharmacological pain-relief and childbirth experience may be partially confounded by prenatal education.

## 4. Discussion

This study explored intrapartum services associated with women’s childbirth experience. A total of 498 women from ten tertiary facilities in mainland China completed the survey. Network analysis results indicated that several intrapartum services were associated with childbirth experience and may warrant consideration in quality-improvement planning, alongside clinical guidelines, feasibility, resources and women’s preferences.

Women in this study reported relatively a high level of childbirth experience, with a mean score slightly higher than that reported in a previous Chinese study [24]. For the childbirth experience, our respondents reported that they were provided with the most professional support, felt highly secure and were well cared for. This study was conducted in ten tertiary hospitals from urban areas with comparatively substantial clinical resources, which may partly explain the relatively high childbirth experience scores.

Nevertheless, some CEQ-C dimensions indicated scope for improvement. The most obvious gap for improvement observed in this study was women’s self-perception of the childbirth experience, which included feeling tired and scared, which is consistent with previous studies [25,26]. A Spanish study similarly found that women reported fear and tiredness despite feeling secure and professionally supported [25]. These findings suggest that even when high-level professional support is available, self-perception, such as fear or tiredness, remains the lowest-scoring dimension among Chinese women in tertiary-hospital settings, which deserves clinical attention. The fear probably comes from concern for the safety of the fetus [27], idealistic expectations of the childbirth process, and a lack of professional information obtained during the antenatal period [28]. Therefore, it is important to recognize the endeavour women undertake during childbirth and to reduce their potential fear of childbirth through the provision of professional antenatal information and the establishment of a realistic expectation of childbirth.

In this cross-sectional sample, network analyses showed associations between childbirth experience and intrapartum services. The results showed that women who received a greater number of evidence-based intrapartum services during the first and the second stages of labour reported more positive childbirth experiences. It is notable that a greater number of intrapartum services being received represents broader exposure to recommended care bundles, yet service quality, implementation fidelity and the preference-match remain critical dimensions, and could not be captured by this numerical count alone. Additionally, other evidence indicated that the number of obstetric interventions was associated with a stepwise higher prevalence of negative birth experience, which should be taken into consideration [29].

Using the correlational analysis approach, it can be inferred that, among the prioritized intrapartum services, SSC showed the strongest association with higher childbirth experience scores among women. WHO recommended naked, immediate and uninterrupted SSC for at least 90 min [2]. Previous studies on SSC mainly focused on its effects on breastfeeding outcomes [30,31]. We found that women who had immediate and naked SSC for a longer duration had a more positive childbirth experience, which has not been reported previously to the best of our knowledge. In addition, a gap between the recommended 90 min SSC duration and the average duration of 36 min was observed in this study. When interpreted alongside WHO guidance [30], immediate and uninterrupted naked SSC for at least 90 min may warrant consideration in quality-improvement initiatives for various clinical practice settings.

Based on the combined results from the Bayesian networks and random forest analyses, we observed that the use of a pharmacological pain-relief method was an important bridging factor, connecting women’s demographic characteristics and their childbirth experience. Evidence indicated that the relationship between pain-relief methods and childbirth experience could be complex [32] and could be confounded by multiple maternal or obstetrics factors [33]. For instance, multiparous women had a better childbirth experience than primiparous women [26]. Primiparous women’s childbirth experience has been associated with epidural analgesia, duration of labour, and oxytocin augmentation [33]. In our study, the use of non-pharmacological pain-relief methods was associated with a more positive childbirth experience, consistent with previous research [33]. A retrospective cohort study of 85,448 women showed that primiparous women with epidural analgesia had higher risk of a negative childbirth experience when compared with those who were given non-pharmacological pain-relief [33]. Women should therefore receive balanced information about available pharmacological and non-pharmacological options and be supported in making informed choices.

The above statistical differences in effect sizes deserve explicit consideration of their clinical significance. In the present study, most of the effect sizes ranged from weak to moderate, with SSC indicators yielding moderate-to-large effect sizes. For multi-factorial patient-reported outcomes, such as childbirth experience, large correlation coefficients are rarely expected because maternal perception is jointly shaped by psychological traits, expectations, obstetric complications and service factors. Moderate-magnitude associations in this study suggest these modifiable intrapartum services represent practically relevant targets for quality improvement. Notably, given the nature of the cross-sectional study design, these effect sizes reflect associations rather than causal effect magnitudes.

Notably, a few evidence-based intrapartum services did not rank highly as influential factors of women’s childbirth experience, such as free position or mobility in the first stage of labour. Although most of the women were encouraged to adopt a free position (85%) in this study, most of them were still in a supine (57%) or semi-recumbent position (30%). This could be the reason why no statistically significant connections between ‘position during labour’ and women’s childbirth experience were observed. Meanwhile, women reported low participation in choosing their position during labour although they acknowledged that they had the right to take free position during childbirth. This suggested that more efforts are required, such as updating facilities’ clinical routine and eliminating barriers in facilitating evidence-based strategy, to further enhance women’s experience during childbirth. Another evidence-based strategy, continuous midwifery care, did not rank high in this study. Less than half of the women were provided care by the same midwife before and after childbirth in this study and less than half of the women mentioned their midwife’s name. This situation was in line with the fact that a continuous midwifery model of care is not currently widely adopted in the Chinese mainland, which could be the reason why this evidence-based strategy was not prioritized by women in relation to their childbirth experience in this study.

### 4.1. Strengths and Limitations

This study has several strengths. Consistent with the global WHO guidelines, our study demonstrates that SSC, multi-modal pain-relief and full implementation of staged evidence-based labour services were positively associated with women’s childbirth experience. However, the incremental novelty of this work is reflected in two analytical outputs derived from the random forest and Bayesian network. Firstly, we quantified the weight of each intrapartum service and ranked the top-priority interventions under limited clinical resources. Secondly, we mapped the interactive network of maternal characteristics, various intrapartum services and four dimensions of childbirth experience, identifying pharmacological pain-relief as the core bridging node linking population characteristics and birth experience, and grouping services into four key functional communities that jointly shape women’s perception of childbirth. Such visualized network-based quantitative priority evidence can provide complementary insights. Finally, the multi-site sampling of hospitals captured diversity among urban tertiary hospitals across different economic regions of mainland China, including both general and maternal-specialized tertiary facilities.

Admittedly, this study has its limitations. Firstly, as observational cross-sectional data, all detected relationships are associations rather than causal effects, and causal inference cannot be established. Secondly, although we applied cross-validation and bootstrap procedures to mitigate model overfitting, the generalizability of the specific variable importance rankings may be influenced by the current sample characteristics. Thirdly, the investigated facilities were mostly urban tertiary hospitals located in high- and middle-resource regions, with relatively sufficient medical staffing, equipment and midwifery service resources. The priority ranking of intrapartum services observed in this study cannot be directly generalized to rural, primary or low-resource regions. Fourthly, although reviewing medical notes ensured the objectiveness and reliability of data collection for most variables, SSC-related services and experiences were self-reported by women, which may lack precision and introduce common method bias. In future studies, triangulation with clinical records and perceptions from healthcare providers should be incorporated. Fifthly, this study applied time-stratified quota sampling rather than probability-based sampling. No random starting point or fixed sampling interval was used during participant selection, which may introduce selection bias. Although we covered all three daily birth time intervals to improve representativeness, the findings should be generalized with caution. Sixthly, while we used interaction terms rather than stratified models to preserve statistical power for subgroup analyses, we acknowledge that small cell sizes in certain subgroups may limit the precision of subgroup-related estimates. Finally, we recommend including qualitative insights in future studies to explore the potential effects of unmeasured demographic/obstetric confounders, such as cultural beliefs and providers’ attitudes, on women’s childbirth experience. Residual confounding due to these unmeasured factors cannot be fully ruled out. Additionally, centre-level clustering was not formally modelled; future multilevel or cluster-robust approaches may further account for this source of variation.

### 4.2. Implications for Nursing Clinical Practice

Using network-analysis approaches, the implications of intrapartum services were explored in this study. Firstly, women who are supported with a greater number of evidence-based intrapartum services have a better childbirth experience, especially services during the second stage of labour. This suggests that facilities should ensure an extensive array of evidence-based services to ensure women can make a broader scope of informed choices, such as supporting a woman to choose and change their position during labour. Secondly, SSC service and pain-relief methods should be provided with optimal priority. Uninterrupted SSC services immediately after childbirth, naked and for a longer duration, for example, 90 min, were shown to be related to a positive experience during childbirth in this study. In addition, pharmacological and non-pharmacological pain-relief methods should be offered based on women’s options and preferences to improve their perception of their own involvement and increase positive experiences.

It should be noted that the above recommendations are based on observations from tertiary hospital settings. For low-resource settings with limited manpower and infrastructure, step-by-step strategies are proposed. For first-tier priority, low-cost, zero-equipment interventions could be promoted, including immediate naked SSC, continuous labour companionship and spontaneous pushing guidance. For the second-tier priority, low-investment non-pharmacological pain-relief options could be introduced, such as birth balls, massage, and breathing guidance. Pharmacological pain-relief options could be planned as long-term targets when staffing and funding permit. Note that the priority ranking in this study only refers to the predictive contribution weight of each service to childbirth experience scores from statistical models. Actual clinical implementation priority requires integrated consideration of human resource costs, facility feasibility and resource consumption.

## 5. Conclusions

Different from prior studies that merely verify the effectiveness of single WHO-recommended services, this study innovatively adopts an integrated network analysis to quantitatively prioritize intrapartum services and reveal their complex interaction pathways. Network analysis results indicated that compared with women’s demographic characteristics and obstetric outcomes, the evidence-based intrapartum services women received during labour presented the strongest positive correlation with overall CEQ-C childbirth experience scores. Longer, uninterrupted, naked SSC, the combined provision of non-pharmacological and pharmacological pain-relief options, and full delivery of labour services corresponded to higher birth experience scores in this sample.

For clinical priority recommendations synthesized from this study together with WHO guidelines, the implementation of immediate, uninterrupted, naked SSC for at least 90 min, diversified pain-relief options, and the supply of full-process evidence-based services throughout the first and second stages of labour should be prioritized. More evidence from low-resource areas is needed to verify whether the same priority ranking of intrapartum services applies to low-resource clinical contexts, providing targeted tiered strategies for various clinical practice settings.

## Figures and Tables

**Table 1:** Basic information of study sample (demographic variables and obstetric outcomes).

Item	Content	n	%	Item	Content	n	%
Maternal age	<28 years	165	33.1	Prenatal education	Yes	180	36.1
	28–34 years	249	50.0		No	318	63.9
	≥35 years	84	16.9	Parity	Primiparous	248	49.8
Educational level	Below undergraduate	162	32.5		Multiparous	250	50.2
	Undergraduate	219	44.0	Maternity leave	Yes	411	82.5
	Bachelor and above	117	23.5		No	87	17.5
Household register for the couple	Both urban household register	251	50.4	Status on hospital admission	Waiting for labour	169	33.9
	Both rural household register	125	25.1		Others	329	66.1
	One urban household register	122	24.5	Birth mode	Spontaneous vaginal birth	487	97.8
Occupational types	Employed	319	64.1		Forceps or vacuum	11	2.2
	Not employed	179	35.9	Perineal laceration	Intact	114	22.9
Family annual income	<120 thousands RMB	65	13.1		Not intact	384	77.1
	≥120 thousands RMB	433	86.9	Perineal suture	No suture	26	5.2
Complication	Yes	270	54.2		Suture	472	94.8
	No	228	45.8	Gender of newborn	Male	271	54.4
Pregnancy risk level	Low risk (green)	227	45.6		Female	227	45.6
	Moderate risk (yellow)	244	49.0	Blood loss (total)	<500 mL	448	90.0
	High risk (orange)	27	5.4		≥500 mL	50	10.0
Routine examination during pregnancy	Routine times of examination (12–13 times)	454	91.2	Time to initiate breastfeeding	Within 30 min after childbirth	312	62.7
	Less than routine times of examination	7	1.4		After 30 min after childbirth	186	37.3
	More than routine times	23	4.6	Duration of the first-time breastfeeding	0–29 min	329	66.1
	Unclear	14	2.8		30–59 min	129	25.9
					60 min and above	40	8.0

**Table 2:** Scores for each item and dimension of the Childbirth Experience Questionnaire (Chinese version).

Dimension	Item Content	Mean	SD
1 Professional Support		3.64	0.49
	13 My midwife devoted enough time to me.	3.58	0.60
	14 My midwife devoted enough time to my partner.	3.51	0.68
	15 I was kept informed.	3.67	0.53
	16 My midwife understood my needs.	3.66	0.54
	17 I felt very well taken care of by the midwife.	3.66	0.56
	18 I felt secure.	3.75	0.44
2. Own Capacity		3.24	0.65
	1 Labor progress went as I had expected.	3.52	0.74
	2 I felt strong.	3.31	0.80
	4 I felt capable.	3.22	0.77
	6 I felt happy.	3.00	0.91
	7 I have many positive memories.	3.07	0.90
	19 The situation was well handled.	3.34	0.81
3. Self-Perception		2.70	0.70
	3 I felt scared. ^R^	2.43	0.99
	5 I felt tired. ^R^	1.97	0.92
	8 I have many negative memories. ^R^	3.19	0.90
	9 I felt depressed. ^R^	3.23	0.89
4. Participation		3.06	0.79
	10 I could choose whether to be up and moving or lying down.	3.26	0.89
	11 I could choose the delivery position.	2.82	1.04
	12 I could choose the pain relief method.	3.11	0.92

^R^: Ratings of negatively worded statements are reversed. SD: Standard deviation.

**Table 3:** Comparing characteristics for tertiary maternal hospitals and tertiary general hospitals.

Variable	Cramér’s V	Test Method	*p*_Value	*p*_Adjusted
Initiation time of SSC	0.468	Chi-square	<0.001	<0.001
Type of SSC	0.410	Chi-square	<0.001	<0.001
Childbirth experience	0.352	Chi-square	<0.001	<0.001
Pharmacological pain-relief method	0.344	Chi-square	<0.001	<0.001
Duration of SSC	0.245	Chi-square	<0.001	<0.001
Non-pharmacological pain-relief method	0.230	Chi-square	<0.001	<0.001
Company during the first and second stage of labour	0.195	Chi-square	<0.001	<0.001
Household register for the couple	0.173	Chi-square	0.001	0.002
Complicate	0.165	Chi-square	<0.001	0.001
Multipara	0.161	Chi-square	<0.001	0.001
Perineal suture	0.159	Chi-square	<0.001	0.001
Services during the second stage of labour	0.158	Fisher	0.002	0.006
Initiation time of breastfeeding	0.158	Chi-square	0.002	0.005
Educational level of women	0.146	Chi-square	0.005	0.011

**Table 4:** Ten highest-ranked factors identified by the random forest drop-out method.

Ranking	Name of the Service	Abbreviations	Transformed Random Forest Values	Intrapartum Service
1	Duration of SSC	SSC.Duration	0.94	Yes
2	Services during the second stage of labour	S2.Service	0.87	Yes
3	Type of SSC	SSC.Type	0.84	Yes
4	Initiation time of SSC	SSC.InitialT	0.67	Yes
5	Services during the first stage of labour	S1.Service	0.58	Yes
6	Non-pharmacological pain-relief method	Nonpharmacy	0.39	Yes
7	Initiation time of breastfeeding	BF. InitialT	0.26	No
8	Company during the first and second stage of labour	S12.Company	0.24	Yes
9	Duration of the first breastfeeding	BF.InitialD	0.22	No
10	Complication during pregnancy	Complicate	0.20	No

**Table 5:** Associations between intrapartum services and CEQ-C scores based on Spearman’s rank correlation.

Item		Overall CEQ-C	CEQ-1	CEQ-2	CEQ-3	CEQ-4
Services during the second stage of labour	S	0.36	0.30	0.29	0.22	0.35
	95%CI	−0.27–0.45	0.22–0.38	0.20–0.37	0.13–0.31	0.26–0.42
	*p*	<0.001 **	<0.001 **	<0.001 **	<0.001 **	<0.001 **
Services during the first stage of labour	S	0.35	0.32	0.29	0.19	0.31
	95%CI	0.27–0.43	0.23–0.39	0.21–0.36	0.10–0.28	0.24–0.40
	*p*	<0.001 **	<0.001 **	<0.001 **	<0.001 **	<0.001 **
Initiation time of SSC	S	0.32	0.31	0.25	0.20	0.23
	95%CI	0.24–0.40	0.23–0.40	0.17–0.34	0.12–0.28	0.14–0.31
	*p*	<0.001 **	<0.001 **	<0.001 **	<0.001 **	<0.001 **
Duration of SSC	S	0.30	0.24	0.20	0.26	0.25
	95%CI	0.22–0.39	0.16–0.33	0.11–0.28	0.18–0.34	0.16–0.34
	*p*	<0.001 **	<0.001 **	<0.001 **	<0.001 **	<0.001 **
Type of SSC	S	0.29	0.28	0.25	0.19	0.19
	95%CI	0.20–0.37	0.20–0.36	0.16–0.34	0.11–0.27	0.1–0.27
	*p*	<0.001 **	<0.001 **	<0.001 **	<0.001 **	<0.001 **
Non-pharmacological pain-relief methods	S	0.17	0.11	0.13	0.14	0.16
	95%CI	0.08–0.25	0.02–0.20	0.05–0.22	0.06–0.23	0.07–0.24
	*p*	0.001 **	0.015 *	0.003 *	0.001 *	<0.001 **
Pharmacological pain-relief methods	S	0.10	0.11	0.05	0.10	0.06
	95%CI	0.01–0.19	0.02–0.21	−0.04–0.14	0.02–0.19	−0.03–0.14
	*p*	0.031 *	0.013 *	0.269	0.022 *	0.205

Note: *: *p* < 0.05; **: *p* < 0.001; S: Spearman coefficient; CI: confidence interval.

## Data Availability

The data that support the findings in this study are available from the corresponding author on reasonable request.

## Supplementary Materials

The following supporting information can be downloaded at: https://www.mdpi.com/article/10.3390/healthcare14183115/s1, Material S1: Time-stratified quota sampling for women’s survey; Figure S1: Flowchart of recruitment of participants; Figure S2: The relationship of SSC duration with women’s childbirth experience; Table S1: Services received during childbirth reported by women; Table S2: Prenatal education subgroup analysis for evaluating confounding influence on impact of Skin-to-skin contact type on childbirth experience; Table S3: Prenatal education subgroup analysis for evaluating confounding influence on impact of Skin-to-skin contact initiation time on childbirth experience; Table S4: Prenatal education subgroup analysis for evaluating confounding influence on impact of Skin-to-skin contact duration on childbirth experience; Table S5: Prenatal education subgroup analysis for evaluating confounding influence on impact of pharmacological pain relief on childbirth experience; Table S6: Prenatal education subgroup analysis for evaluating confounding influence on impact of non-pharmacological pain relief on childbirth experience; Table S7: Prenatal education subgroup analysis for evaluating confounding influence on impact of free mobility in the first stage of labour on childbirth experience; Table S8: Prenatal education: Confounding and effect modification analysis.
