# Supplementary material for: Exploring Priority Intrapartum Services Associated with Childbirth Experience During Vaginal Birth: A Multicenter Cross-Sectional Study
Source: Healthcare (Basel). 2026 Sep 21;14(18):3115. doi: 10.3390/healthcare14183115 (PMC13606815; doi:10.3390/healthcare14183115)

Time-stratified quota sampling for women's survey

Investigator checks <Childbirth Record Book> in the birth center for all the births in the previous day. Based on the inclusion criteria, investigators count the eligible women to expected amount. If the amounts of vaginal birth in the previous 24 hours was no more than 7 cases, all the cases were included for the survey; If the amounts was more than 7 cases, select 2 or 3 cases from each duration of the 24 hours (duration 1: 0:00 am-7:59 am; duration 2: 8:00 am-17:59 pm; duration 3: 18:00 pm-23:59 pm). This strategy repeats until the expected number of survey was completed.

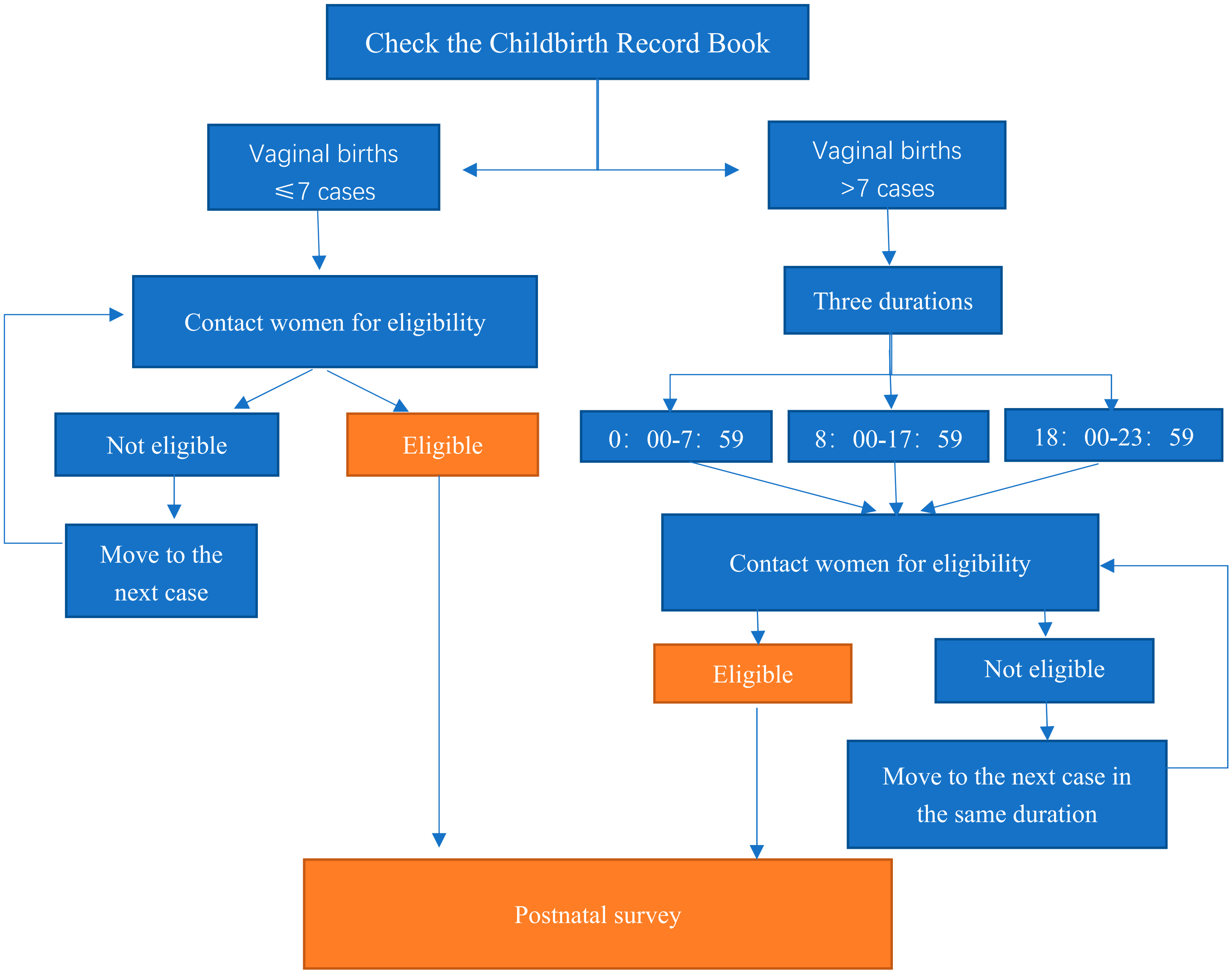

Supplement: Supplementary file 1 [file healthcare-14-03115-s001.zip › Proofreading-Supplementary Material 1-Sampling procedure.pdf]
