# Supplementary material for: Exploring Priority Intrapartum Services Associated with Childbirth Experience During Vaginal Birth: A Multicenter Cross-Sectional Study
Source: Healthcare (Basel). 2026 Sep 21;14(18):3115. doi: 10.3390/healthcare14183115 (PMC13606815; doi:10.3390/healthcare14183115)
Supplement: Supplementary file 1 [file healthcare-14-03115-s001.zip › Proofreading-Supplementary Material 2-Supplementary tables and figures.pdf]

Supplementary Material 2-Supplementary tables and figures

Supplementary Table S1. Services received during childbirth reported by women (N=498)

Item	Content	n	%
Acknowledge of midwife’s name	Yes	221	44.4
	No	116	23.3
	Can’t remember	161	32.3
The same midwife before and during labour	Yes	206	41.4
	No	183	36.7
	Unclear	109	21.9
Companion in the first stage of labour	Yes	420	84.3
	No	78	15.7
Companion during the second stage of labour (except medical staff)	Yes	259	52.0
	No	239	48.0
Free mobility in the first stage of labour	Yes	404	81.0
	No	94	18.9
Permission to eat and drink in the first stage of labour	Yes	464	93.2
	No	34	6.8
Permission to eat and drink during the second stage of labour	Encouraged or permitted	435	87.3
	Not required	63	12.7
Reminder to urinate in the first stage of labour	Yes	457	91.8
	No	41	8.2
Provision of non-pharmacy pain relief method	Yes	328	65.9
	No	114	22.9
	Unclear	56	11.2
Pharmacy pain relief	Yes	338	67.9
	No	158	31.7
	Unclear	2	0.4
Pharmacy pain relief methods	Epidural analgesia	322	95.3
	Others	16	4.7

Item	Content	n	%
Free position during the second stage of labour	Yes	423	84.9
	No	75	15.1
Position adopted mostly during the second stage of labour	Supine	284	57.0
	Semi-recumbent	150	30.1
	Lateral position	31	6.2
	Use birth stools	30	6.0
	Others	3	0.6
Spontaneous pushing during the second stage of labour	Yes	166	33.3
	No	332	66.7
Fundal compressions	Yes	210	42.2
	No	288	57.8
Induction	Yes	124	24.9
	No	374	75.1
Augmentation of labour	Yes	158	31.7
	No	340	68.3
Type of skin-to-skin contact	Yes, naked	355	71.3
	Yes, with clothes	61	12.2
	No	82	16.5
Time to initiate skin-to-skin contact	Immediately after birth within one minute	228	45.8
	Within five minutes after birth	125	25.1
	After five minutes after birth	63	12.7
The duration of skin-to-skin contact	Less than 30 minutes	207	49.8
	30-59 minutes	87	20.9
	60-89 minutes	28	6.7
	90 minutes and above	94	22.6

Supplementary Table S2. Prenatal education subgroup analysis for evaluating confounding influence on impact of Skin-to-skin contact type on Childbirth experience.

Prenatal education	Skin-to-skin contact type	Childbirth experience score level Q1/3 n(%)	Q2/3 n(%)	Q3/3 n(%)	*P*-Value
No	Naked	6 (2.6%)	84 (36.7)	139 (60.7%)	<.001
	No	5 (9.8%)	24 (47.1%)	22 (43.1%)	
	With clothes	4 (10.5%)	24 (63.2%)	10 (26.3%)	
Yes	Naked	4 (3.2%)	38 (30.2%)	84 (66.7%)	<.001
	No	7 (22.6%)	16 (51.6%)	8 (25.8%)	
	With clothes	2 (8.7%)	15 (65.2%)	6 (26.1%)	

Supplementary Table S3. Prenatal education subgroup analysis for evaluating confounding influence on impact of Skin-to-skin contact initiation time on Childbirth experience.

Prenatal education	Skin-to-skin contact Initiation Time	Childbirth experience score level Q1/3 n(%)	Q2/3 n(%)	Q3/3 n(%)	*P*-Value
No	<1min	2 (1.3%)	52 (34.9%)	95 (63.8%)	<.001
	<5min	4 (4.7%)	40 (47.1%)	41 (48.2%)	
	>5min	4 (12.1%)	16 (48.5%)	13 (39.4%)	
	No	5 (9.8%)	24 (47.1%)	22 (43.1%)	
Yes	<1min	2 (2.5%)	18 (22.8%)	59 (74.7%)	<.001
	<5min	1 (2.5%)	17 (42.5%)	22 (55.0%)	
	>5min	3 (10.0%)	18 (60.0%)	9 (30.0%)	
	No	7 (22.6%)	16 (51.6%)	8 (25.8%)	

Supplementary Table S4. Prenatal education subgroup analysis for evaluating confounding influence on impact of Skin-to-skin contact duration on Childbirth experience.

Prenatal education	Skin-to-skin contact duration	Childbirth experience score level Q1/3 n(%)	Q2/3 n(%)	Q3/3 n(%)	*P*-Value
No	<30 min	12 (6.4%)	91 (48.4%)	85 (45.2%)	.003
	30-59 min	3 (5.6%)	19 (35.2%)	32 (59.3%)	
	60-89 min	0 (.0%)	8 (44.4%)	10 (55.6%)	
	90 and above	0 (.0%)	14 (24.1%)	44 (75.9%)	
Yes	<30 min	13 (12.9%)	50 (49.5%)	38 (37.6%)	<.001
	30-59 min	0 (.0%)	11 (33.3%)	22 (66.7%)	
	60-89 min	0 (.0%)	2 (20.0%)	8 (80.0%)	
	90 and above	0 (.0%)	6 (16.7%)	30 (83.3%)	

Supplementary Table S5. Prenatal education subgroup analysis for evaluating confounding influence on impact of Pharmacy pain relief on Childbirth experience.

Prenatal education	Pharmacy pain relief	Childbirth experience score level Q1/3 n(%)	Q2/3 n(%)	Q3/3 n(%)	*P*-Value
No	No	6 (6.0%)	48 (48.0%)	46 (46.0%)	.163
	Yes	9 (4.1%)	84 (38.5%)	125 (57.3%)	
Yes	No	6 (10.0%)	27 (45.0%)	27 (45.0%)	.176
	Yes	7 (5.8%)	42 (35.0%)	71 (59.2%)	

Supplementary Table S6. Prenatal education subgroup analysis for evaluating confounding influence on impact of Non-pharmacy pain relief on Childbirth experience.

Prenatal education	Non-pharmacy pain relief	Childbirth experience score level Q1/3 n(%)	Q2/3 n(%)	Q3/3 n(%)	*P*-Value
No	More than one method	1 (1.0%)	42 (40.0%)	62 (59.0%)	.014
	No	11 (8.3%)	62 (47.0%)	59 (44.7%)	
	One method	3 (3.7%)	28 (34.6%)	50 (61.7%)	
Yes	More than one method	5 (5.3%)	33 (34.7%)	57 (60.0%)	.285
	No	4 (10.5%)	19 (50.0%)	15 (39.5%)	
	One method	4 (8.5%)	17 (36.2%)	26 (55.3%)	

Supplementary Table S7. Prenatal education subgroup analysis for evaluating confounding influence on impact of Free mobility in the first stage of labour on Childbirth experience.

Prenatal education	Free mobility in the first stage of labour	Childbirth experience score level Q1/3 n(%)	Q2/3 n(%)	Q3/3 n(%)	*P*-Value
No	No	3 (6.0%)	24 (48.0%)	23 (46.0%)	.478
	Yes	12 (4.5%)	108 (40.3%)	148 (55.2%)	
Yes	No	5 (20.0%)	14 (56.0%)	6 (24.0%)	.001
	Yes	8 (5.2%)	55 (35.5%)	92 (59.6%)	

Supplementary Table S8. Prenatal education: Confounding and Effect Modification Analysis

Variable	Beta_crude	Beta_adjusted	SE_crude	SE_adjusted	P_crude	P_adjusted	Beta_change_pct	Interaction_P
SSC.Duration	0.975	0.994	0.623	0.624	0.117	0.111	1.976	0.198
SSC.Initialtime.Type	-0.847	-0.857	0.680	0.680	0.212	0.207	1.244	0.471
SSC.type	-1.777	-1.772	0.447	0.448	0.000	0.000	0.274	0.490
Nonphamacy	-1.140	-1.346	0.494	0.511	0.021	0.008	18.011	0.425
Pharmacy	0.489	0.481	0.394	0.395	0.214	0.222	1.610	0.810

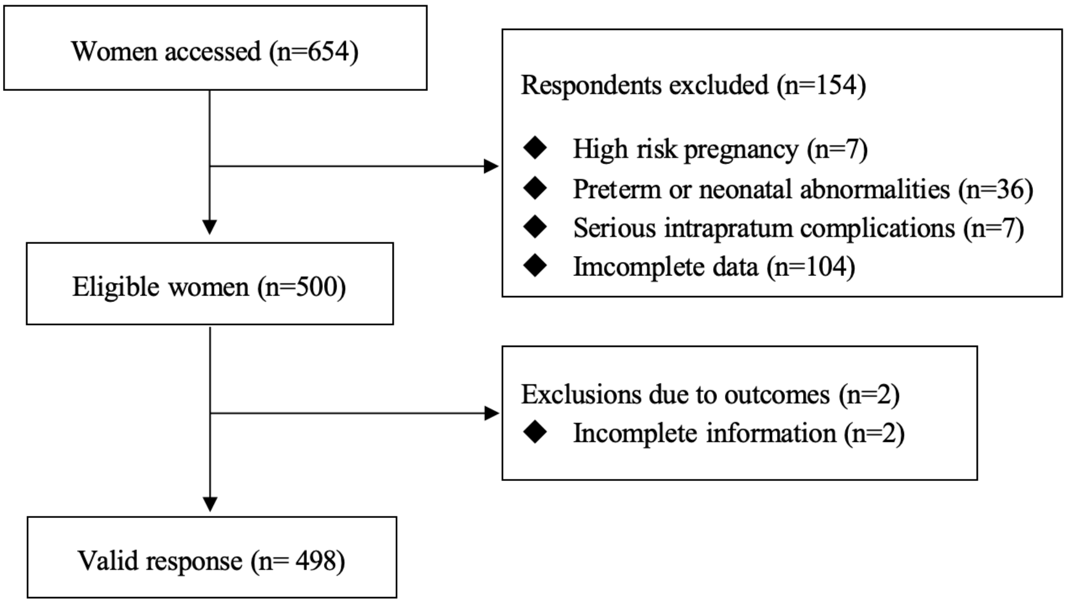

Supplementary Figure S1. Flowchart of recruitment of participants

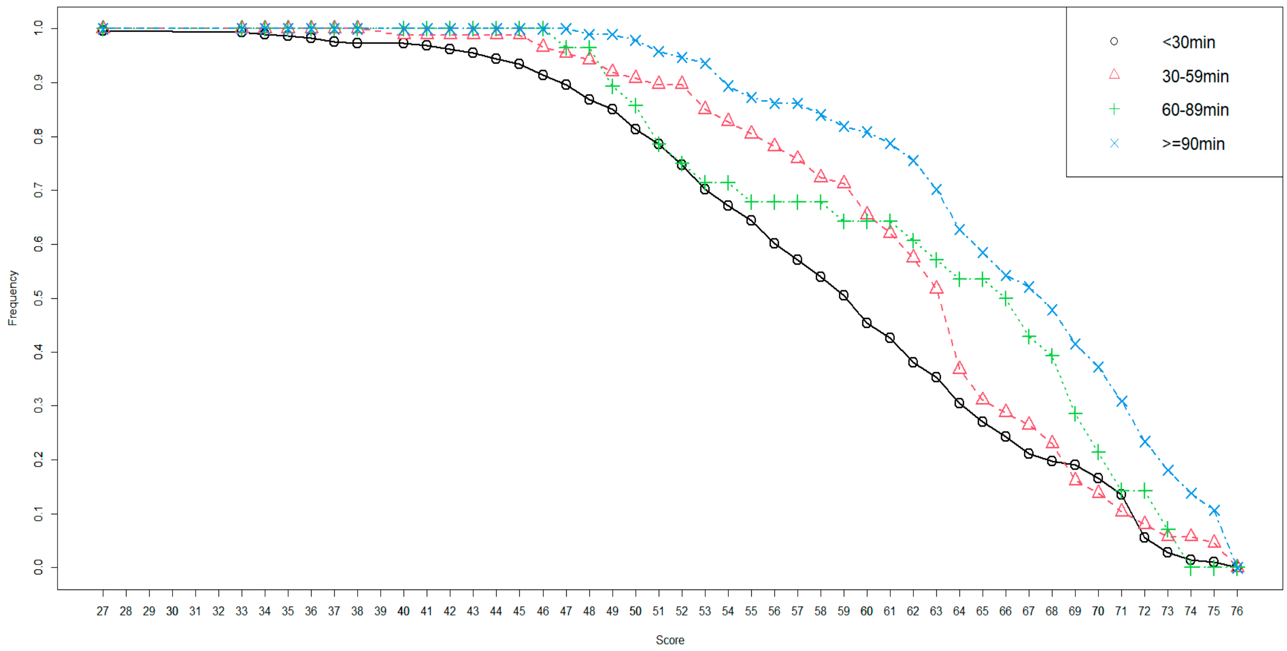

Supplementary Figure S2. The relationship of SSC duration with women's childbirth experience.
